# Nutrition quality of food purchases varies by household income: the SHoPPER study

**DOI:** 10.1186/s12889-019-6546-2

**Published:** 2019-02-26

**Authors:** Simone A. French, Christy C. Tangney, Melissa M. Crane, Yamin Wang, Bradley M. Appelhans

**Affiliations:** 1 Division of Epidemiology and Community Health, School of Public Health, University of Minnesota, 1300 South Second Street, Suite 300, Minneapolis, MN 55454 USA; 2 Department of Clinical Nutrition, College of Health Sciences, Rush University Medical Center, 600 Paulina Street, Room 716, Chicago, IL 60612 USA; 3 Department of Preventive Medicine, Rush University Medical Center, 1700 W Van Buren Street, Suite 470, Chicago, IL 60612 USA; 4 Department of Internal Medicine, Rush University Medical Center, 1645 W. Jackson, Suite 675, Chicago, IL 60612 USA

**Keywords:** Food purchases, Household income, Nutritional quality, Dietary intake

## Abstract

**Background:**

Lower household income has been consistently associated with poorer diet quality. Household food purchases may be an important intervention target to improve diet quality among low income populations. Associations between household income and the diet quality of household food purchases were examined.

**Methods:**

Food purchase receipt data were collected for 14 days from 202 urban households participating in a study about food shopping. Purchase data were analyzed using NDS-R software and scored using the Healthy Eating Index 2010 (HEI 2010). HEI total and subscores, and proportion of grocery dollars spent on food categories (e.g. fruits, vegetables, sugar sweetened beverages) were examined by household income-to-poverty ratio.

**Results:**

Compared to lower income households, after adjusting for education, marital status and race, higher income households had significantly higher HEI total scores (mean [sd] = 68.2 [13.3] versus 51.6 [13.9], respectively, adjusted *p* = 0.05), higher total vegetable scores (mean [sd] = 3.6 [1.4] versus 2.3 [1.6], respectively, adjusted *p* < .01), higher dairy scores (mean [sd] = 5.6 [3.0] versus 5.0 [3.3], *p* = .05) and lower proportion of grocery dollars spent on frozen desserts (1% [.02] versus 3% [.07], respectively, *p* = .02).

**Conclusions:**

Lower income households purchase less healthful foods compared with higher income households. Food purchasing patterns may mediate income differences in dietary intake quality.

**Trial registration:**

ClinicalTrials.gov identifier: NCT02073643.

## Background

Low income is associated with a poor quality dietary intake [1, 2]. Compared to those with higher income, lower income individuals consume fewer fruits and vegetables, more sugar-sweetened beverages and have lower overall diet quality [1, 2].

Household food purchases are important to examine because they provide information about potential mediators of individual dietary intake, and have implications for intervention strategies to improve dietary intake and quality. Individual dietary intake is shaped in part by the household food purchases that create the home food environment [3, 4]. Household food purchase receipt collection provides detailed, timely data on the type and cost of foods and beverages flowing into the home environment [5]. Food purchase receipt data have been used to examine specific food categories of interest, nutrients and overall healthfulness of the home food environment. Low-income households purchase fewer fruits and vegetables, more sugar-sweetened beverages and fewer healthful foods compared with higher income households [4–14].

The purpose of the present research was to examine differences in the quality of food purchased by household income level. Data are from an observational study of food shopping practices that included 202 urban households in a large city in the United States [15]. It was hypothesized that lower income households’ food purchases would be lower in overall nutritional quality, and include fewer fruits and vegetables and more sugar-sweetened beverages compared with higher income households. A unique aspect of the present study was its examination of specific types of foods purchased and overall nutritional quality using the Healthy Eating Index 2010 [16], and its inclusion of purchases from a variety of food stores, not just traditional grocery stores [5, 6, 17].

## Methods

### Study population and recruitment

The sample was composed of Chicago households enrolled in the Study of Household Purchasing Patterns, Eating, and Recreation [SHOPPER] [15], a cross-sectional study of behavioral and socioeconomic correlates of food purchasing patterns [ClinicalTrials.gov identifier: NCT02073643]. A convenience sample was recruited from the community between 2014 and 2016 through posted flyers, newspaper advertisements, mailings, craigslist.org, word-of-mouth, and other methods. Interested individuals completed a telephone screening to assess eligibility. Adults who reported making ≥75% of their household’s food purchases were eligible to participate. Exclusion criteria included: 1) non-fluent in English, 2) not living in Chicago, 3) major food allergies or sensitivities, 4) religious/spiritual or medical dietary restrictions that could impact food choice, and 5) living in temporary or group housing or living with a roommate with whom food is shared. Of 347 households screened, 300 (86.5%) met eligibility criteria and 209 (69.7%) enrolled. Five participants were withdrawn from the study because of scheduling conflicts that arose during the 14-d assessment period (*n* = 3) or due to noncompliance with the protocol (*n* = 2). Two additional participants were not included in the analysis reported here because food receipts were not returned to the research team. The final analysis sample included 202 participants. Participants were compensated $100 for completing all four assessments. Written informed consent was obtained from all participants. Study procedures were approved by the Rush University Medical Center Institutional Review Board.

### Measures

#### Food purchases and receipt collection

Participants were trained by research staff to collected their food purchase receipts and complete annotation procedures throughout the 14-d measurement period. Research staff visited participants’ homes four times during the 14-d measurement period to collect food purchase receipts from participants, with phone calls between to enhance adherence to the food purchase receipt protocol. The receipt data collection protocols are adapted from our previous research studies [5, 7, 15, 17–19].

The primary household food shopper was trained to collect and annotate food purchase receipts from all household members on a daily basis (even for purchases without a receipt). Annotation sheets were completed by the participant that included the date, time, source and location, payment methods and foods purchased, including item quantity, size, price, and brief description. Color coded stickers were applied by the participant to both the receipt annotation sheet and the food packages. Food packages were saved for research staff to have direct access to the nutrition information. Details about foods without packaging or nutrition labeling (e.g., fresh produce, deli items, bulk nuts/candy) were recorded by researchers during each of the four data collection home visits. Research staff queried participants about any foods purchased that were consumed immediately and therefore had neither receipts nor food packages with nutrition information (e.g., carry-out or restaurant meals). Ready-to-eat foods that could not be accurately characterized (e.g., a buffet meal purchased and consumed by a household member other than the primary shopper) were deemed “non-codable” and were not subjected to nutrient analysis (< 1% of all purchases).

#### Food purchase nutrient analysis and diet quality

The Nutrition Data System for Research (NDS-R) [20], was used to compute the nutritional analysis of household food purchases. NDS-R is a database that contains nutrient information for over 18,000 foods and is constantly updated for accuracy and to include newly available foods. The Healthy Eating Index-2010 scoring system [16] was used to compute the diet quality of the food purchase data once entered into the NDS-R software system. The HEI-2010 scores the nutrient densities (kcal/g, per 1000 kcal) for 12 key dietary components on a continuous scale based on conformity to the Department of Health and Human Services’ 2010 Dietary Guidelines for Americans [1]. The 12 component scores are summed to obtain a total score with a maximum of 100 points, with higher scores reflecting better overall diet quality. HEI sub-scores examined here included the following: total fruit; whole fruit; total vegetables; greens and beans; whole grains; dairy; total protein foods; seafood and plant proteins; fatty acids; refined grains; sodium; empty calories. The following food groups created by the NDS-R food coding system were also examined as a second method to describe the quality of the household food purchases: 1) fruits; 2) vegetables; 3) sugar-sweetened beverages (SSBs); 4) sweet baked items; 5) packaged snack foods; 6) frozen desserts; 7) other desserts; 8) candy. The dollars spent on each food category was divided by the total dollars spent from grocery and other stores (excluding restaurants). Of the 2342 receipts collected, 1349 (57%) were from stores and 993 were from eating out or other sources. Only receipts from food stores were included in the analysis of dollars spent.

#### Demographic and social variables

The primary shopper self-reported age, gender, ethnicity/race, educational attainment, employment, marital status, household size and composition and household income. The income-to-poverty ratio was calculated by dividing annual household income by the current Federal Poverty Threshold [21], which accounts for the number of adult and child family members in the household.

### Statistical analyses

The analytic sample includes 202 subjects with complete food purchase, diet recall, and sociodemographic data. Analyses were performed using SAS 9.4 (Cary, NC). Descriptive statistics were calculated to characterize the study sample and food purchasing variables. The food purchase variables derived from the receipt data include the HEI-2010 scores and component scores, and dollar amount spent within pre-specified food categories. These values were calculated for all food purchases combined. However, for the dollars spent variables, purchases from restaurant / eating out sources were excluded due to the inability to determine prices for foods and beverages purchased as a combination (e.g., meals including an entrée, side and beverage with a single price). Models were examined using a three-level category of income-to-poverty ratio as the independent variable. Cutpoints were selected based on values previously used for national data [21]: Low: 0–1.3 (*n* = 49); Medium: 1.4–3.4 (*n* = 78); High: > 3.5 (*n* = 74). High income-to-poverty ratio indicates higher income. Adjusted models were examined that included covariates that might be associated with food spending: education, race and marital status. Unadjusted and adjusted models are shown in the tables below. Results were considered statistically significant where *p* < .05.

## Results

### Descriptive variables by income

Participants in the sample were primarily female, with a varied distribution on household size, children in the household, education, race, marital status and other variables (Table 1). Significant differences by income were observed for most demographic and household variables. Those with lower income were less likely to be married, had larger household size, were more likely to have obesity, be African American, have a high school education or less, not be employed full time, and be currently enrolled in SNAP.

**Table 1:** Demographic and Household Variables by Income-to-Poverty Ratio

	Income-to-Poverty Ratio			
	Low 0–1.3	Medium 1.4–3.4	High 3.5+	Unadjusted p
*N* = 202	49	78	75	
Demographic Variables (%;n)				
Sex (% female, n females)	83.7 (41)	82.1 (64)	84.0 (63)	.94
Age (yrs)				.32
18–29 (*n* = 31)	6.1 (3)	19.2 (15)	17.3 (13)	
30–49 (*n* = 96)	53.1 (26)	47.4 (37)	44.0 (33)	
50+ (*n* = 74)	40.8 (20)	33.3 (26)	38.7 (29)	
Household Size				.03
1	34.7 (17)	34.6 (27)	36.0 (27)	
2	24.5 (12)	20.5 (16)	42.7 (32)	
3	8.2 (4)	10.3 (8)	5.3 (4)	
4+	32.7 (16)	34.6 (27)	16.0 (12)	
Children in Household (yes)	42.9 (21)	51.3 (40)	81.3 (61)	.0001
Marital Status				.002
Single	42.9 (21)	34.6 (27)	25.3 (19)	
Cohabitate/married	18.4 (9)	44.9 (35)	54.7 (41)	
Divorced/separated/widow	38.8 (19)	20.5 (16)	20.0 (15)	
Weight Status				.0001
normal weight	12.2 (6)	25.6 (20)	45.3 (34)	
overweight	8.2 (4)	23.1 (18)	25.3 (19)	
obese	79.6 (39)	51.3 (40)	29.3 (22)	
Race				.0001
African American (*n* = 87)	77.6 (38)	48.7 (38)	20.0 (15)	
Latino/Other (*n* = 50)	16.3 (8)	30.8 (24)	24.0 (18)	
White (*n* = 60)	6.1 (3)	20.5 (16)	56.0 (42)	
Education				
< High school	28.6 (14)	11.5 (9)	1.3 (1)	
Some college	55.1 (27)	39.7 (31)	14.7 (11)	
College degree	12.2 (6)	37.2 (29)	37.3 (28)	
≥ College degree	4.1 (2)	11.5 (9)	46.7 (35)	
Employed Full Time	12.2 (6)	37.2 (29)	69.33 (52)	.0001
Food Secure	22.5 (11)	41.0 (32)	60.0 (45)	.0002
SNAP enrolled	83.7 (41)	46.2 (36)	6.7 (5)	.001

Note: Percents (N) are unadjusted

### Nutrition quality of food purchases

#### HEI scores

Nutrition quality of food purchases by income is shown in Table 2. Healthy Eating Index 2010 scores were significantly associated with income in both unadjusted (*p* < .0001) and adjusted (*p* = .05) analyses. In post hoc comparisons, HEI total scores were significantly lower among low-income compared with high-income households (*p* = .03, in adjusted analyses). No significant differences were observed between low- and medium-income households after adjustment for education, marital status and race (*p* = .58).

**Table 2:** Healthy Eating Index 2010 Scores for Food Purchases by Income-to-Poverty Ratio

	Income-to-Poverty Ratio^a^			Unadjusted	Adjusted	Adjusted High v. Low	Adjusted Medium v. Low
	Low 0–1.3	Medium 1.4–3.4	High 3.5+				
*N* = 202	49	78	75				
Food Purchases HEI-2010 total	51.6 (13.9)	57.8 (15.1)	68.2 (13.3)	.0001	.05	.03	.58
HEI-fruit total	1.6 (1.7)	2.1 (1.6)	3.1 (1.6)	.0001	.08	.11	.94
HEI - whole fruit	1.9 (1.9)	2.6 (1.8)	3.7 (1.7)	.0001	.19	.12	.74
HEI - veg total	2.3 (1.6)	2.6 (1.5)	3.6 (1.4)	.0001	.01	.01	.57
HEI - green/bean	2.0 (2.2)	2.4 (2.1)	3.4 (1.8)	.0003	.37	.33	.94
HEI - whole grain	3.3 (3.4)	4.3 (3.5)	5.0 (3.7)	.03	.91	.71	.93
HEI - refined grain	6.5 (3.5)	6.5 (3.6)	7.8 (2.9)	.03	.10	.10	.95
HEI - dairy	5.0 (3.3)	5.0 (2.9)	5.6 (3.0)	.38	.05	.02	.06
HEI total protein	4.0(1.4)	4.4 (1.1)	4.2 (1.2)	.27	.19	.17	.07
HEI-seafood/plants	2.0 (2.0)	2.8 (2.0)	3.4 (2.0)	.001	.14	.05	.11
HEI-fatty acids	5.1 (3.2)	5.1 (3.5)	5.1 (3.6)	.99	.99	.87	.92
HEI-sodium	5.1 (4.5)	6.1 (3.7)	6.8 (3.7)	.05	.07	.02	.14
HEI-empty calories	12.9 (5.7)	14.0 (5.4)	16.4 (4.2)	.0003	.43	.32	.97

NOTE: ^a^Unadjusted means and standard deviations are shown in table

Adjusted = adjusted for race, marital status and education

In unadjusted analyses, most HEI sub-scores significantly differed by income group, and the pattern was that lower-income households had lower (poorer nutrition quality) scores compared with higher-income households. In analyses adjusted for education, marital status and race, there were significant differences by household income for vegetables (*p* = 0.01) and dairy (*p* = 0.05). In both cases, lower income households scored lower than higher income households. No significant differences were observed between lower and middle income household on HEI subscores.

### Proportion of grocery dollars spent

Total dollars spent at grocery and other food stores by income level is shown in Table 3. A positive significant association was observed between income category and dollars spent at grocery and other food stores (*p* < .01). In unadjusted analyses, lower-income households spent a significantly smaller percent of their grocery dollars on fruit (*p* < .003) and vegetables (*p* < .001), and a significantly higher percent of their grocery dollars on sugar sweetened beverages (*p* < .004) and frozen desserts (*p* < .01), compared with higher income households. No significant differences were observed for percent grocery dollars spent for packaged snacks, sweet baked items, other desserts, and candy. The proportion of beverage grocery dollars spent on SSBs was higher among lower income households compared with higher income households (*p* < .0001). In analyses adjusted for education, race and marital status, compared to lower income households, higher income households spent a significantly lower percent of grocery dollars on frozen desserts (*p* = .02). No other income differences were significant after adjustment for education, race and marital status.

**Table 3:** Proportion of Grocery Store Dollars Spent on Food and Beverage Categories by Income-to-Poverty Ratio

	Income-to-Poverty Ratio^a^			Unadjusted p	Adjusted p	High v. Low	Medium v. Low
	Low 0- < 1.3	Medium 1.3 - < 3.5	High 3.5+				
*N* = 202	49	78	75				
Total Grocery Dollars Spent/Week	102.9 (84.0)	141.8 (91.5)	162.4 (108.4)	.005			
Proportion of Grocery Dollars Spent/Week							
Fruit	0.05 (0.07)	0.07 (0.07)	0.10 (0.09)	.003	.27	.24	.98
Vegetables	0.08 (0.07)	0.09 (0.07)	0.13 (0.08)	.001	.06	.19	.60
Sugar Sweetened Beverages	0.06 (0.06)	0.05 (0.07)	0.02 (0.06)	.004	.95	.95	.85
Sugar Sweetened Beverages/Total Beverages	0.56 (0.34)	0.40 (0.36)	0.22 (0.34)	.0001	.68	.38	.59
Packaged Snacks	0.07 (0.09)	0.05 (0.07)	0.05 (0.06)	.16	.26	.11	.17
Sweet Baked Items	0.03 (0.04)	0.05 (0.09)	0.04 (0.12)	.51	.39	.47	.18
Other Dessert	0.00 (0.01)	0.00 (0.00)	0.00 (0.00)	.29	.15	.06	.09
Frozen Dessert	0.03 (0.07)	0.01 (0.03)	0.01 (0.02)	.01	.02	.01	.01
Candy	0.05 (0.07)	0.03 (0.06)	0.02 (0.07)	.14	.32	.17	.17

NOTE: Of the 2342 receipts collected, 1349 (57%) were from stores and 993 were from eating out or other sources. Only receipts from food stores were included in the analysis of dollars spent

^a^Unadjusted means and standard deviations

Adjusted = adjusted for marital status, race and education

## Discussion

Household food purchases are important to examine because they may influence dietary intake quality, and are important potential intervention targets. In the present study, the overall nutritional quality of foods and beverages purchased was significantly lower among lower income households compared with higher income households. This remained significant with adjustment for education level, a strong correlate of both household income and diet quality. The specific food purchase categories that were associated with income were vegetables and dairy (HEI subscores) and frozen desserts (NDS-R food category). Vegetable purchases coded into the HEI subcategories were significantly positively associated with higher income-to-poverty ratio, and were marginally associated with purchases measured by NDS-R food categories coding. Dairy purchases, captured by the HEI subcategories, and frozen desserts, captured by the NDS-R food categories, significantly differed by income-to-poverty ratio.

The results of the present study are consistent with existing data regarding the association between income level and the nutritional quality of foods and beverages purchased [6–14]. Food purchase data show that lower-income households purchase less healthful foods overall, fewer fruits and vegetables and more sugary beverages compared to households with higher income [6–14]. The most recent comprehensive analysis of food purchase patterns from a nationally representative sample of 4826 US households showed that food purchase patterns among households of all income levels are lower in dietary quality than recommended [4]. However, households that were participating in the federal food assistance program (called Supplemental Nutrition Assistance Program) purchased lower quality foods compared to households of comparable income that were not participating, and households with higher income. Overall Healthy Eating Index scores, fruits, vegetables and whole grains were significantly lower and empty calories significantly higher, among low-income households enrolled in SNAP compared with low-income households not enrolled in SNAP and higher income households [4]. In another study, an analysis of 24,879 household food purchase receipts showed that food purchases by lower-income households were less healthful and included fewer fruits and vegetables than recommended, according to a standardized nutrient profile [11]. In another study of 90 households with children, compared with higher income households, lower income households spent fewer dollars on fruits and vegetables and sweets and snacks, but spent a larger proportion of beverage dollars on sugary beverages [7]. A study of 1003 households that used face to face interviews found that lower income households reported purchasing fewer fruits, vegetables and fiber, and more sugary foods, compared with higher income households [9, 10]. In a study that used in-store shoppers’ purchase data, results showed that lower-income household purchases were lower in dietary quality per 1000 kcals purchased compared with higher income households’ food purchases [8].

These findings further establish the link between income and the quality of the foods and beverages purchased by households. If diet quality is lower among lower-income groups, then food purchases may be a key intervention target. The present study indicates that lower income households are less likely to purchase recommended healthful foods such as vegetables, and spend a larger proportion of their grocery money on less healthful foods such as frozen desserts. Food assistance programs could help promote healthier food purchases through specific program guidelines, such as incentivizing the purchase of fruits and vegetables, or restricting the purchase of sugar-sweetened beverages or sweet baked goods [19, 22]. These strategies have been shown to be effective in changing low-income households’ food purchases in community-based randomized trials [18, 19, 22].

The present study was limited in its ability to separately examine income and education in relationship to food purchasing behavior. Income and education are closely intertwined, and may have independent or synergistic effects on food purchasing behaviors. It is noteworthy that many of the observed associations between income and food purchasing variables were substantially attenuated when adjusting for other socioeconomic variables such as education and race. The independent effects of education and income on food purchases warrants closer study, since intervention strategies may be differentially effective, depending on the answers to these questions.

The use of receipts to measure household food purchases has methodological limitations, including lack of information about the completeness of the receipts to represent all food purchases during the time interval covered [5, 7, 23]. No objective measure exists of the true total number of receipts that participants should turn in to the research staff. Thus, it is not known whether participants turned in 100, 50% or some other portion of their total food purchase receipts. It is possible that participants may have omitted receipts for small purchases such as a single drink or candy item [5, 7]. By contrast, a strength of the receipt data is its potentially lower reactivity than self-report assessments. It is an objective measure of food purchases, does not rely on participant memory, and may be less affected by social desirability responding. The enrolled sample was comprised of volunteers, and this could affect the generalizability of the results reported here.

Lower quality food purchasing among lower-income households may be due to higher food prices for higher quality foods [3, 21–25]. Even within lower-income households, higher quality food purchases are associated with spending more money on those particular food categories [3, 24]. Household configuration and the presence and number of children, and employment-related variables, including number of jobs and hours worked, may also influence the quality of foods and beverages purchased. Future research should examine the influence of these variables on the quality of household food and beverage purchases using large cohorts that will enable adequately powered analysis of these demographic and household variables.

## Conclusions

Lower income households purchase foods of lower nutritional quality compared to higher income households. Lower nutritional quality of foods purchased could contribute to the lower diet quality observed among lower income individuals. Further research is needed to understand how the nutritional quality of foods purchased can be improved on a limited income.

## Abbreviations

HEI: Healthy Eating Index
SSB: Sugar sweetened beverages
